# Perceptions of Therapeutic Climbing for Patients with Multiple Sclerosis in Neurorehabilitation: A Qualitative Study

**DOI:** 10.3390/healthcare12060674

**Published:** 2024-03-17

**Authors:** Tobias Schiffler, Eva Reiter, Ali Kapan, Gottfried Kranz, Stefan Thomas Kotzian, Sandra Haider

**Affiliations:** 1 Center for Public Health, Department of Social and Preventive Medicine, Medical University of Vienna, Kinderspitalgasse 15/1, 1090 Vienna, Austria; tobias.schiffler@meduniwien.ac.at (T.S.);; 2 Department for Clinical Neurosciences and Preventive Medicine, University for Continuing Education Krems, Dr.-Karl-Dorrek-Straße 30, 3500 Krems, Austria; 3 Neurological Rehabilitation Center Rosenhügel, Rosenhügelstraße 192a, 1130 Vienna, Austria; gottfried.kranz@nrz.at (G.K.); stefan.kotzian@nrz.at (S.T.K.)

**Keywords:** multiple sclerosis, therapeutic climbing, motivation, training conditions, neurorehabilitation, qualitative research

## Abstract

Background: Therapeutic climbing (TC) has emerged as a prospective rehabilitation approach for individuals with multiple sclerosis (MS). The existing literature primarily focuses on the physical and psychological benefits of TC across diverse populations but is limited concerning its application and efficacy for patients with MS. Objectives: This study aimed to delineate the experiences, subjective effects, and perceptions of both individuals with MS and therapists regarding TC, highlighting the potential benefits and challenges of this therapeutic approach. Methods: Using a qualitative design, semi-structured interviews were conducted with patients living with MS (N = 5) and therapists (N = 7) involved in TC sessions at a rehabilitation facility. The interviews were recorded, transcribed verbatim, and subjected to thematic qualitative text analysis. Results: Our analysis resulted in the identification of five main categories: (1) motivational factors, (2) training conditions, (3) training content, (4) observed effects, and (5) safety protocol. Our findings primarily centred around the motivational aspects of TC. Participants consistently reported experiencing feelings of accomplishment, success, enjoyment, and increased self-confidence. Furthermore, TC was often perceived as a comprehensive intervention, addressing endurance, strength, flexibility, neuromotor functions, cognition, and mental health while having a low-risk profile. However, due to the demanding nature of TC, careful fatigue management is crucial. This entails personalised intensity adjustments during sessions and coordinating TC with other physically demanding therapies when implementing TC within a rehabilitation environment. Conclusions: TC shows promise within MS rehabilitation and can be considered safe under certain framework conditions. This research sheds light on its potential benefits, facilitators, and barriers and provides insights for practical integration into rehabilitation programs.

## 1. Introduction

Multiple sclerosis (MS), also known as encephalomyelitis disseminata, is an inflammatory neurodegenerative disease affecting the central nervous system, which includes the brain and spinal cord [1,2]. This chronic autoimmune condition typically manifests early [3], frequently resulting in long-term disabilities and diminished social participation [4,5], as well as cognitive impairments and concentration problems [6,7]. The symptoms of MS can be managed through medications and non-drug therapies, such as physiotherapy, occupational therapy, speech therapy, psychotherapy, neuropsychological therapy, and psychosocial care [8].

Among non-drug therapies, therapeutic climbing (TC) has emerged as a unique and innovative approach for treating patients with MS. It involves non-cyclical whole-body movements that integrate elements of strength and endurance training [9,10], differing from traditional therapies by offering a holistic physical challenge that engages both the mind and body in a dynamic and stimulating environment. The versatility of TC, ranging from bouldering without a safety rope to tailored exercise regimens and rope climbing [9,11,12,13], allows for adaptability to suit individual patient needs and preferences, a key advantage over more conventional therapeutic modalities.

Originally utilised in orthopaedics, TC’s application has broadened to include neurology, psychiatry, and psychology [12], demonstrating its versatility and effectiveness across a wide range of conditions. To date, numerous studies have highlighted the benefits of climbing on healthy individuals, citing improvements in balance, coordination, core stability, mobility, and muscle strength, all while carrying minimal risk of injury [14,15,16,17]. Further, a systematic review [18] and a narrative review [12] underscored the potential of TC to alleviate MS symptoms, enhancing physical fitness, fatigue management, self-efficacy, and overall quality of life. Delving deeper into specific studies, a randomised controlled trial with 20 participants compared sport climbing, using a belt and a top rope system, to yoga [19]. After ten weeks, participants in the climbing group observed a 25% decrease in the severity of their reported disabilities and a 33% decline in fatigue levels. Another RCT (n = 27) found a positive impact on fatigue after two hours of TC weekly over six months [13].

Despite the growing body of evidence supporting the benefits of TC for various populations, research specifically addressing its use and efficacy for people with MS in rehabilitation settings remains limited. This gap underscores the need for comprehensive research to explore the benefits and challenges of implementing TC for patients with MS in such environments. In response to this identified need, our study aimed to delve into the detailed experiences related to TC for patients with MS within a rehabilitation environment. We examined various facets, including motivational factors, necessary training conditions, observed effects, and safety measures. Thus, barriers and facilitators toward the relatively novel intervention of TC should be uncovered. From the perspectives of both patients and therapists, we sought to understand how and why certain elements hinder or promote the use of TC and to understand the unique aspects of TC that foster its use over other approaches. By highlighting TC’s distinctive benefits, this research aimed to provide insights into optimising TC’s therapeutic efficacy for patients with MS.

## 2. Materials and Methods

### 2.1. Study Design

In adopting a qualitative research methodology, this manuscript was written according to the COREQ guidelines (Supplementary Materials) [20]. This study was performed at a public rehabilitation facility in Vienna hosting 147 inpatient and 30 outpatient therapies specialising in neurological and neuropsychological rehabilitation [21]. The primary aim of this facility is to either restore or sustain patients’ functional and performance abilities through maintaining an integrated multidisciplinary approach, with a team comprising physiotherapists, training therapists, dieticians, occupational therapists, speech therapists, neuropsychologists, orthoptists, nursing staff providing bedside therapeutic interventions, and neurologists. While TC is recognised as a standard intervention within the rehabilitation framework, its application is tailored to individual patients based on specific goal settings, indications, and contraindications. Sessions, typically lasting 25 min, are conducted 2 to 3 times weekly. During each session, two patients engage in climbing activities on two neighbouring boulder walls, each spanning a height of 2.5 m. One therapist diligently oversees the activities throughout these sessions to ensure appropriate patient safety and guidance.

In terms of the assignment of individuals to the TC group, the multidisciplinary team evaluated each patient’s condition and rehabilitation needs to determine their suitability for TC. This assessment involved considering the patient’s physical capabilities, potential benefits from engaging in TC, and any specific health concerns that might contraindicate participation in such activities. This process ensured that TC was not only aligned with the therapeutic objectives, but also adapted to the unique needs and safety considerations of each patient.

During this study, we applied several techniques to meet the primary quality criteria regarding the trustworthiness of our qualitative data. In terms of credibility, we sought peer debriefs from colleagues experienced in qualitative research to provide us with their external perspectives to validate our interpretations. To enhance the dependability of the data, we maintained an audit trail in the form of a detailed record of our research process. Furthermore, we ensured consistency in our data collection methods through predetermined protocols. For confirmability, we strengthened our team’s reflexivity through regular discussions to acknowledge and mitigate our biases. Finally, we contextualised our findings and interpretations in this work to allow transferability to other settings [22].

### 2.2. Participants

**Figure 1:**
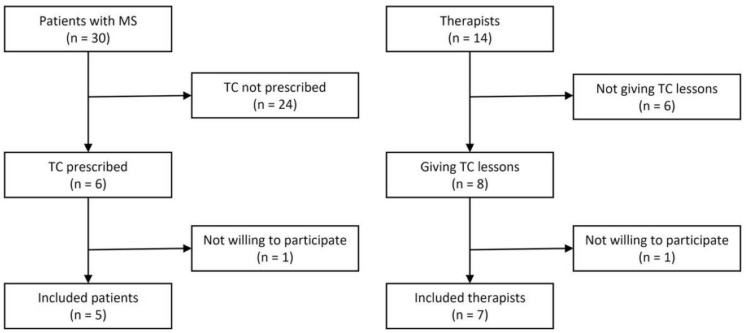
Flowchart of participants. MS: multiple sclerosis; TC: therapeutic climbing.

Two groups of participants were included, namely patients with MS and therapists working with them at the rehabilitation facility. To ensure representativity, all potential participants at the rehabilitation facility were approached face-to-face to inquire about their interest in participating in the study. From 25 April 2022 to 21 July 2022, all patients and therapists meeting the eligibility criteria were invited to partake in qualitative interviews. The inclusion and exclusion criteria were established as follows. Patients had to be (1) diagnosed with MS according to ICD-10 (code: G35) [1], (2) aged ≥ 18 years, (3) undergoing rehabilitation at the facility where the research was conducted, and (4) assigned to TC, as well as (5) provide informed consent. Patients who did not possess sufficient knowledge of the German or English language were excluded. Therapists were required to (1) be sport teachers or sport scientists working at the rehabilitation facility, (2) possess a minimum of one year of practical experience in TC with patients with MS, and (3) provide informed consent (Figure 1).

### 2.3. Data Collection

To gather individuals’ subjective experiences, feelings, perspectives, and attitudes, two group-specific sets of semi-structured in-depth interview guidelines were employed for patients and therapists (see File S1 for the full interview guidelines) [23]. For both groups, interview schedules comprised training modalities, motivational aspects, subjective effects, safety considerations, and the applicability of the therapeutic approach, with group-specific sub-questions. Additionally, therapists were asked about criteria that would lead to the exclusion of patients from TC. Both interview guidelines were pretested with a medical professional experienced in sport climbing and slightly adapted subsequently. Furthermore, interview schedules were refined in comprehensive discussion rounds between researchers after each interview. Due to a lack of time and resources, we opted against a repeat-interview design. However, the guidelines were iteratively refined based on preliminary feedback from the initial interviews, indirectly incorporating participant perspectives into their development.

All interviews were conducted individually and face-to-face at the premises of the rehabilitation facility by one physiotherapist (E.R.). When this study was undertaken, she was an active physiotherapist at the rehabilitation facility, with five years of professional experience. Before the study commenced, no previous relationship existed between her and the included patients. Participants were informed of the interviewer’s professional role within the rehabilitation facility and the research objectives before their interview. Patient interviews were scheduled at the earliest in the second week of their rehabilitation, ascertaining they had acquired sufficient experience with TC. Therapists were interviewed during their available working hours, ensuring they had a one-hour window without any patients. Interviews lasted between 24 and 36 min, and field notes were made during or after every interview. Interview transcripts were not returned to participants for further comments or corrections.

In addition to the interviews, patients’ age, sex, duration of illness, and Expanded Disability Status Scale (EDSS) scores were collected. The EDSS is a scale ranging from 0 to 10 that provides information on an individual’s level of disability, with higher values indicating more severe impairments [24]. People scoring between 1.0 and 4.5 have a high degree of ambulatory ability, whereas those scoring between 5.0 and 9.5 experience a decline in their ambulatory ability. Additional information collected from therapists includes sex, years of work, and TC experience.

### 2.4. Data Analysis

**Figure 2:**
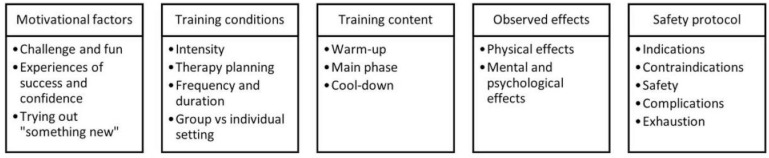
Overview of adapted code set categories.

The interviews were audio-recorded, manually transcribed, and analysed using thematic qualitative text analysis following Kuckartz’s methodology [25]. Initially, two researchers (E.R. and T.S.) separately coded data collected from patients and therapists. T.S. is a nursing practitioner specialised in mental health with expertise in qualitative research methods and evaluating therapeutic interventions. By assigning relevant text sequences to major codes derived from the two interview guidelines, the researchers adopted a deductive approach, yielding two group-specific code sets that were refined and expanded throughout the process. Iteratively comparing and contrasting the tentative codes with the original transcripts enhanced the precision and explanatory capacity of the subsequent inductive text analysis. Based on the collected interview material, the two researchers inductively adapted and condensed the initial code sets, which resulted in six main categories with corresponding subcategories in a single code set (Figure 2). In order to reach a consensus during the process, the two coding researchers employed a systematic and iterative approach with regular in-person or online meetings to discuss their individual progress and coding decisions, resulting in a shared codebook. If a disagreement between coders occurred, triangulation was employed by introducing a third independent researcher from the study team (S.H.) to review and mediate these discrepancies. Participants were not further involved in this stage. Both the transcription and coding were conducted software-aided utilising MAXQDA 2022.

### 2.5. Ethical Considerations

The Ethics Committee of the University for Continuing Education, Krems, granted ethical approval for this study (EK GZ 08/2021-2024). Participants provided informed consent both in verbal and written form prior to their individual interview session. The interview transcripts were pseudonymised to ensure data privacy by assigning consecutive numbers as codes to replace participants’ names. While patients and therapists were allocated an individual number within their groups, patients’ codes were designated with an additional letter “P”, and therapists’ codes were indicated with “T”. Participants were assigned their codes after signing the informed consent form. Consequently, only the study authors could identify the individuals involved. Once the verbatim transcription was completed, all audio files were utterly deleted.

## 3. Results

At the rehabilitation facility, 30 patients with MS and 14 therapists were available during the study period. TC was not prescribed to 24 patients by their physicians in charge because the therapy did not align with their individual goals. In the group of therapists, six were not offering TC and were therefore excluded from participation. Furthermore, two eligible individuals, one patient and one therapist, chose not to take part in the study. Eventually, a total of 12 participants, comprising 5 patients and 7 therapists, were interviewed (Figure 1). Three female and two male patients aged 35 to 59 participated in the study (Table 1). The duration of their disease varied between 1 and 24 years. The patients’ median EDSS score was 3.0, with a minimum of 2.0 and a maximum of 3.5. All patients engaged in TC sessions and attended them between 3 and 11 times.

Among therapists, there were three women and four men. The therapists’ experience with TC ranged from 2 to 15 years (Table 2).

The final code set consisted of the five main categories: (1) motivational factors, (2) training conditions, (3) training content, (4) observed effects, and (5) safety protocol, with subcategories indicated in Figure 2.

### 3.1. Motivational Factors

All participating patients associated TC with a sense of **challenge and fun**, as they embraced the difficulties of the tasks, finding joy in overcoming them, and perceived these experiences as driving factors. Concurrently, all interviewed therapists emphasised the motivational aspect of fun emerging during TC sessions, recognising its positive influence on patients’ engagement in the entire therapeutic process:

*And the big advantage is that the exercises are very short, and then you immediately have a success–and if you have no success, then you think to yourself that it also does not matter. […] But it is simple, uplifting. It is good for the mind. It raises your self-esteem again, and that’s a fun factor, where you know: “Yes, if I make it, then it’s good, and if not, it doesn’t matter”.*

(P2)

In addition, patients consistently reported their experience of achieving a profound level of concentration that seamlessly transitioned into a state often referred to as “flow”:

*“You have quite a tunnel vision, then. For me, it is like this. I do not notice much around me. I am also quite sensitive to noise, but I am highly concentrated. I get into a flow and always want to do things right. So, I do not always want to do things halfway–I just want to do it right”.*

(P3)

Related to the desired outcome and among various motivational factors mentioned by both patients and therapists, the exhilaration of pushing one’s physical limits and **experiences of success and confidence** stood out prominently. Several respondents emphasised the intense engagement and focus that climbing demanded, which contributed significantly to their motivation:

*“The motivation is to get it done. So certain movements, whether the basic position or coming up [the wall], reach as high as I can. The nice thing is that you see the result immediately. If it works or does not work, that is just great. And if it does not work, I just do it again. So that is really special, and I really enjoy it”.*

(P5)

*“I liked that although I am thin, I have quite a bit of strength in my hands, and I can use that. I have a good feeling because I know: “You can do that”. And I am not at the mercy of the feeling that I am going to fail”.*

(P3)

Respondents enormously appreciated the training content, which they perceived as highly effective or remarkably diverse. As a means to further ends, the allure of **trying out “something new”** and the vast array of activities were seen as additional motivational factors. This sentiment was echoed by three professionals who highlighted TC’s capability to introduce patients with MS to novel experiences. They saw it as an opportunity to present patients with a potential new hobby. Two therapists valued TC’s application versatility and capacity to provide patients valuable feedback through heightened body awareness during climbing.

### 3.2. Training Conditions

The success of TC depends on several conditions, including intensity, frequency and duration, and setting (individual or group). All respondents unanimously described training **intensity** as ranging from intensive to very intensive:

*“And that is simply what distinguishes it from physiotherapy, where it is quite clear what is happening, for instance, squatting down or with the heels on the floor–just as great. It is just that with climbing, there is also the aspect of fun, while at the same time, it is somehow very intense, but that is good”.*

(P5)

P5 further elaborated on the unique challenges posed by climbing:

*“When you face challenges in climbing, you can’t just take it lightly. In other forms of training, you can adjust the weight to your preference; for instance, I could choose to train with 5 kilos. But in climbing, you’re contending with your own body weight. That’s the weight you must manage”.*

Moreover, the patients perceived TC as demanding and strenuous due to the constant requirement of supporting one’s body weight during exercises. However, this high training intensity was indicated as an inherent aspect of TC that is thus inevitable but can be modulated to fit patients’ needs:

*“Because as a therapist, it starts relatively soon that I play with the intensities. If I see that the person is doing a great job and it is working great, I can think about doing a more difficult exercise for them. If I see that the exercise is already quite demanding, then I can make the next exercise easier”.*

(T2)

Therapists highlighted challenges in **therapy planning**, emphasising the need to coordinate TC sessions with other physically demanding therapies like strength training or physiotherapy. Moreover, they stressed that it is advisable not to schedule TC on two consecutive days. This particular observation was also echoed by the patients themselves, who often expressed the desire for a break immediately after a TC session:

*“I just have to rest for a short while after the training. […] Half an hour or hour and then I feel very fit again”.*

(P4)

All patients expressed satisfaction with the **frequency and duration** of the therapy sessions, deeming them appropriate. They acknowledged that longer therapy sessions might lead to a potential loss of concentration and increased frustration:

*“Longer [sessions] would avail to nothing because then you get into exhaustion, and then you are demotivated, because it does not work. So, because it is also a strength exercise, with this trunk stabilization, I think that is a good time interval. Just that long, not longer, not shorter”.*

(P2)

Patients and therapists responded in controversial ways regarding the TC **group vs. individual setting**. However, several advantages of the group setting were highlighted by both groups. Firstly, patients expressed their enjoyment of training in pairs, finding it a pleasurable experience. Secondly, the presence of fellow participants served as a motivating factor, providing the much-needed incentive to push themselves during the training sessions. Additionally, both groups appreciated the flexibility of taking necessary breaks when required, which the therapists emphasised. Nevertheless, a therapist, T5, highlighted the limitations of the group setting:

*“It is a group, and group programs are usually just not so specific. Or let me put it this way: it is a clear extra effort if you give two patients completely different exercises”.*

(T5)

### 3.3. Training Content

All respondents uniformly divided TC sessions into three main components: a **warm-up** phase, which can take place directly on the climbing wall or the floor, followed by the **main phase** encompassing specific exercises in the basic climbing position and dynamic climbing moves, and finally, an optional **cool-down** phase. All participants positively rated this training structure. The basic climbing position, where both hands and feet maintain contact with the climbing wall, was consistently regarded as a crucial element of TC. This position was seen as instrumental in promoting stability, ensuring safety, and enhancing body awareness during the sessions:

*“So, what I find good is that the exercises always go out from a fixed basic position, where the basic position always recalls this stability again and again. That is–with the shoulders down and abdominal tension and a bit of squatting–heels up”.*

(T1)

Specific exercises, such as targeted grasping, were frequently highlighted for their role in improving coordination. Patients with MS particularly appreciated the activity of climbing from one side of the wall to the other while having to perform tasks. They described this experience as not only providing a pleasant somatic sensation, but also fostering a sense of accomplishment by recognising personal abilities:

*“And today–I climbed today for the second time–I climbed from wall to wall. It was great, both with overhang, it was really cool. […] It is structured great. First, you learn to do the basics and climb that way. […] Then, the arms relaxed, and I actually climbed from right to left and left to right today”.*

(P5)

### 3.4. Observed Effects

In terms of the **physical effects** of TC, four participants reported significant gains in strength, particularly in the trunk, arms, and grip. Therapists also observed several positive physical effects, including improvements in strength, trunk stability, coordination, balance, body perception, mobility, and posture:

*“Of course, [TC is] strengthening the muscles–be it upper arms or grip strength, lower extremities, or trunk stability, respectively. Another effect is, for example, torso stability, spinal stability, posture, but also coordination. And, of course, how to grasp things, how tightly you have to grip in order to be able to hold on. Balance is also trained for people to become a bit more mobile and secure in everyday life and minimize their risk of falling”.*

(T4)

*“In the neck, shoulder, and upper back areas, I notice it already. And, of course, biceps, triceps, you feel very strongly. […] Anyways, you notice the strength, which then just increases a bit. The grip strength is what now just works properly”.*

(P3)

Both therapists and patients reported positive **mental and psychological effects**, including mood, executive functions, social skills, and self-confidence. Notably, self-confidence was highlighted as an area that was particularly empowered, according to the professionals:

*“I think climbing also has a high motivational character, so the patients gain self-confidence and security. By climbing not only at standing height but also a little higher, I believe that patients gain self-confidence and thus appear more self-assured”.*

(T4)

Likewise, participants with MS mentioned TC’s positive effects on mental health and the benefits of combining physical and cognitive training:

*“I already felt like I fit in. I think I have improved not only my athletic activity but also my cognitive capability because you just have to think ahead: Where do I step? Where do I reach? […] That is not so easy for me, and that is why I actually found it good that I can combine both in one unit, both physically and then mentally a bit”.*

(P3)

### 3.5. Safety Protocol

All therapists unanimously described TC as an excellent whole-body workout. The **indications** for TC primarily revolved around patients’ desire to enhance trunk stability, strength, coordination, and concentration. However, using TC to increase leg muscle strength specifically sparked a controversy among therapists. While one respondent believed in its effectiveness, another therapist expressed reservations, suggesting that TC might not be the ideal method for targeting the legs. Therapists underscored a certain degree of body awareness and coordination as a prerequisite for engaging in TC.

Additionally, they pointed out that patients with MS should possess curiosity and a willingness to try out TC, as this attitude plays a significant role in the effectiveness of the therapeutic approach. Six therapists praised TC for its comprehensive, varied whole-body workout with abundant movement variations. Three of them even drew a comparison with traditional strength training, noting that TC outshined it in promoting body awareness, coordination, and variety:

*“Well, it also has much to do with self-awareness, which is perhaps not the case with normal strength training, where I sit on the machine and simply move the leg press. There is just not as much body awareness as in climbing”.*

(T5)

Several **contraindications** for TC were identified during the therapist interviews, namely severe pain or sensory disorders, the inability to hold onto the climbing wall due to reduced arm function, acute injuries or inflammations, epilepsy, the inability to follow the tasks for 25 min due to limited attention and concentration, severe cardiovascular disease, and the inability to stand safely for 25 min:

*“First of all, they need to have a certain strength in the forefeet–so that they can stand on their forefeet at all–and then hold themselves up with the upper extremities with both hands”.*

(T2)

In addition, patients with MS named paresis, sensory disorders, and severe ataxia as potential contraindications. Three respondents emphasised assuming and maintaining the basic climbing position and necessitating toe stance, sufficient trunk stability, and grip function as essential TC requirements. As a result, patients severely affected by MS or those who had recently experienced a relapse should not be included in TC sessions. Notably, three patients expressed concern about the potential frustration that people with these contraindications might experience if they were assigned to TC despite their conditions:

*“I mean–I have never had the case–but if any extremity would be paralyzed or that somebody has perhaps such feelings of numbness, I imagine that would be difficult. I would not know if that would not be rather frustrating if I felt that way”.*

(P3)

**Safety** was paramount for all patients during the TC sessions, and they unanimously expressed feeling secure throughout the training. The presence of fall mats and spotting (i.e., attentive therapists standing behind them) contributed to creating a protected and reassuring atmosphere:

*“I felt safe because the therapist was always behind me, and I know she catches me when something happens”.*

(P1)

Moreover, therapists themselves rated TC as a very safe therapeutic approach. The low therapist-to-patient ratio, with one therapist attending to two patients, coupled with exercises tailored to the individual abilities of the patients, contributed significantly to ensuring safety during the sessions. Furthermore, using a low jump height, ranging from 30 to 40 cm, was deemed relevant in maintaining a safe environment. Five therapists mentioned spotting as an effective measure to prevent accidents and enhance overall safety:

*“As a therapist, if you notice that it is unsafe, you can also stand directly behind the patients. That means that if they slip, it is safe so that they do not hurt themselves badly”.*

(T1)

Nevertheless, therapists stressed that while regular safety checks of the climbing wall are obligatory to ensure a secure environment, extra caution is necessary during both ascent and descent and when patients move horizontally across the climbing wall.

Participants also raised concerns about potential **complications**, including pain, exhaustion, and overheating. Specifically, four therapists noted that mild pain, particularly in the shoulder area, might occur as a possible side effect:

*“Such [mild] pain may occur again and again, in the shoulders, in the knee joint–but nothing more serious”.*

(T4)

Remarkably, none of the patients reported experiencing pain during or after the climbing sessions. On the contrary, one of them even mentioned reducing their pre-existing back pain as a positive outcome of the TC training.

However, patients also highlighted **exhaustion** as a notable concern. Two respondents specifically mentioned experiencing motor fatigue either during or immediately after climbing. For one of these patients, recovery took a significant amount of time, while the other reported encountering eating restrictions resulting from the exhaustion:

*“Once, it was too much for me. I think this morning was intense because I was training in half-hour intervals without a break–first climbing, then eating. And then, I had an intensity tremor in my hands, and then I noticed […] the trembling of the hands became significantly more, and it was difficult to eat”.*

(P3)

When specifically questioned about fatigue and fatigability, most therapists acknowledged having observed instances of fatigue among their patients with MS. However, they did not recognise it as a major issue of TC:

*“I have to say that I cannot think of any MS patient who has stopped climbing with me because of fatigue. […] When patients stop, it is usually because of pain; those are more likely to be spine patients. […] Of course, what happens from time to time is that they say beforehand that they are totally exhausted. But then you try to arrange it, so the MS patient takes longer breaks”.*

(T5)

Also, one therapist pointed out overheating as a potential complication. To address this concern, the use of cooling vests was suggested.

## 4. Discussion

The present investigation aimed to gain a detailed understanding of the experiences and perceptions of therapists and patients with MS within the context of TC. The results provide important insights into various aspects of TC, including motivational factors, training conditions, training content, observed effects, and safety protocols.

### 4.1. Motivational Factors

In all interviews, motivation—and within this category, challenge and fun in particular—was strongly emphasised by both the participants and therapists. This high motivational factor of TC was also found by Frühauf et al. [10], indicating that the inherent challenges and the fun associated with TC serve as significant motivational factors for individuals participating in such programs. Unlike this study, which examined TC as a general therapeutic modality, our research zeroes in on the application of TC for individuals with MS. Furthermore, by highlighting the motivational dynamics at play, our research underscores the importance of designing therapeutic interventions that not only address the physical and cognitive needs of individuals with MS, but also tap into the motivational drivers that encourage long-term engagement and positive health outcomes.

When looking at the mentioned motivational factors in detail, our findings are aligned with the pre-existing classification defined by Gabler [26]. Accordingly, the motivation to perform sport activities can derive from the engagement in sport activities itself (relating to our subcategory “challenge and fun”), desired outcomes of sport activities (relating to our subcategory “experiences of success and confidence”), and sport activities as a means to further ends (relating to our subcategory “trying out ‘something new’”).

This element appears to play a crucial role in maintaining engagement and adherence—an essential factor for the success of rehabilitative interventions. Considering this high motivational level, TC could potentially encourage long-term physical activity. This is especially important as studies indicate that patients with MS tend to be less physically active than individuals without health issues [27]. The motivational aspects of TC, such as the sense of challenge and enjoyment derived from climbing, could play a pivotal role in altering this trend. A promising pathway for long-term physical activity could involve integration with existing extramural climbing groups specifically tailored for individuals with disabilities. Organisations like the Austrian Alpine Club [28] could lead the way in accommodating patients with MS within their climbing activities. This approach not only ensures a structured and safe training environment, but also capitalises on the communal nature of climbing, potentially enriching the therapeutic experience.

Another innovative approach would be to develop specialised apps that could serve various purposes, from tracking progress and scheduling sessions to providing virtual guidance and support [29,30]. Moreover, such apps could foster a sense of community among users by allowing them to share their achievements, challenges, and tips with others undergoing similar therapeutic processes while integrating gamification elements, such as rewards and challenges, could further enhance engagement, making the rehabilitation process more enjoyable and motivating.

### 4.2. Training Conditions

Our study delineated a structured division of TC sessions into a warm-up phase, a main phase, and an optional cool-down phase, aligning with the literature identifying a well-organised structure as crucial for effective rehabilitation sessions [31]. This setup not only promotes safety, but also ensures a challenging progression and efficient recovery. It is noteworthy that the significance of this structured approach was further validated by our participants themselves, who reported finding the session organisation both effective and fitting to their needs. A fundamental aspect of TC is the emphasis on the basic climbing position. The “four-point contact” approach is particularly considerable, as it trains core stability, a crucial factor for many patients with MS, given their frequent deficits in this area [32]. Core stability is foundational not only for effective movement and balance, but also for performing daily activities more efficiently and safely. By focusing on this fundamental climbing skill, TC provides a direct avenue for enhancing an essential physical attribute that can significantly impact the quality of life for those with MS.

Concerning the intensity, the respondents highlighted TC as a holistic approach and often called it a high-quality “whole-body workout”. The observed intensity, ranging from intensive to very intensive, aligns with the inherent nature of climbing, which demands a higher level of physical exertion [33]. Such intensity is integral to the activity’s effectiveness but also presents a challenge in ensuring that the therapeutic benefits are maximised without compromising patient well-being through overexertion. Therefore, the challenge is to find an optimal balance between intensity and recovery [12,33], especially for patients undergoing a series of therapy sessions [34]. This balance is pivotal in the context of MS, where physical activity must be carefully tailored to accommodate the fluctuating capabilities and limitations of each individual, which is why physical activity recommendations for MS underscore the need for programs that are adaptable to each person’s specific needs and preferences [35,36].

In this respect, a practical strategy for customising the intensity of TC involves the thoughtful scheduling of sessions. Research indicates that many patients with MS feel more energised in the mornings [37]. Thus, aligning intense activities with this period can optimise outcomes, ensuring that patients are at their physical peak and more capable of handling the demands of climbing. Furthermore, gradually increasing intensity can also prevent sudden fatigue [38], the most commonly mentioned issue among interviewed patients with MS. Based on our results, recovery periods post TC sessions are demanded. To effectively manage fatigue while maximising therapeutic benefits, customised therapy plans addressing individual reactions and requirements are essential.

However, it is crucial to address the limitations related to the institutional accessibility and widespread availability of TC. Indeed, while TC offers a holistic and effective approach to rehabilitation, it necessitates specific equipment and specialised training for therapists, which may not be readily available at all rehabilitation facilities. Its implementation is therefore contingent upon the availability of appropriate climbing facilities and the presence of therapists trained in guiding patients through the TC process safely and effectively. Consequently, this requirement can be seen as a significant barrier to the broader adoption of TC as a standard intervention in rehabilitation for patients with MS.

### 4.3. Observed Effects

In looking at the observed effects, the therapeutic benefits of regular physical activity for individuals with MS are well documented, including reduced fatigue, pain, and depressive symptoms and increased functional capacity, balance, muscle strength, and aerobic capacity [39]. These benefits underscore the critical role that physical activity plays in the management and rehabilitation of MS. However, in delving into the observed effects in our study, especially the improvements in strength, trunk stability, coordination, body perception, and psychological aspects, a multifaceted reality of patients’ therapeutic journey is reflected. Previous studies also found possible physiological and psychological benefits from TC where the enhancement of physical attributes such as strength, stability, and coordination through TC not only addresses the direct symptoms of MS, but also contributes to a broader sense of bodily autonomy and control [12,18]. Our current study adds to this discourse by emphasising the combined physical and cognitive challenge TC brings along, requiring problem-solving and strategic planning as participants navigate climbing routes [10,40]. Engaging in such activities can stimulate cognitive functions and potentially mitigate some of the cognitive declines associated with MS. Additionally, the surge in self-confidence reported in our study aligns with previous findings [17], which is a direct consequence of mastering new skills, overcoming challenges, and witnessing personal progress within the TC sessions. Such psychological upliftment is integral to the therapeutic process, offering individuals a stronger sense of agency and capability that can transcend the climbing environment and positively affect other areas of their lives.

### 4.4. Strengths and Limitations

This study has clear strengths in its approach. First, the study was conducted in an appropriate environment—the rehabilitation setting. Especially in this setting, there is very little scientific literature on TC. Moreover, the inclusive participation of both patients with MS and therapists as key respondents enriches the depth and breadth of the data collected. By integrating insights from these two primary stakeholders, the research not only gained a multifaceted perspective, but also bolstered the validity of its findings.

However, this study also has several limitations. Firstly, we could not assess the effects of TC in this setting, as patients were simultaneously involved in other treatments (e.g., physiotherapy, treadmill training). Secondly, some patients had only a few TC sessions, which may have affected the depth of their feedback. Thirdly, the relatively small number of participants from only one institution could limit the breadth of perspectives and experiences captured, potentially affecting the degree of data saturation and transferability of our findings. It is important to note that this limitation was primarily due to the unavailability of more participants at the recruiting facility, as there were no additional eligible individuals at the time of recruitment. This constraint on participant availability inherently restricted our capacity to broaden the participant pool and diversify the insights gathered through our research. Lastly, the potential for interviewer bias in relation to both patient and therapist interviews exists, given that the data collection was conducted by an active physiotherapist at the rehabilitation facility. Although no prior relationship existed between the interviewer and the included patients, the interviewer’s professional role within the facility could have inadvertently influenced participants’ responses.

## 5. Conclusions

Respondents viewed TC positively, appreciating its motivational factor, efficacy, and minimal risk profile. A standout feature of TC is its motivational component, which stems from individuals’ first-hand experiences of enhanced physical capabilities and accomplishments. A finding emerging from this study is the impact of TC on managing and addressing fatigue, a prevalent concern for patients with MS. Feedback from participants indicates that while TC offers tangible physical improvements and boosts motivation, it is crucial to individualise sessions, especially considering the fatigue experienced by patients with MS. To gain a more transparent, more generalisable understanding, future studies should delve deeper, employing quantitative methodologies to evaluate TC’s effectiveness rigorously. Given the intensive nature of TC, meticulous fatigue management and individualised intensity adjustments throughout the therapeutic process are essential.

## Figures and Tables

**Table 1:** Participants’ characteristics—patients with MS.

Code	Sex	Age	Duration of Disease (years)	EDSS Score	TC Units Attended
P1	female	59	22	3.0	3
P2	female	54	24	3.5	6
P3	female	35	11	3.5	11
P4	male	37	5	2.0	7
P5	male	35	1	2.5	6

EDSS = Expanded Disability Status Scale [24]; TC = therapeutic climbing.

**Table 2:** Participants’ characteristics—therapists.

Code	Sex	Work Experience (years)	TC Experience (years)
T1	male	16	4
T2	female	6	3
T3	female	20	15
T4	male	2	2
T5	male	9	9
T6	female	12	2
T7	male	20	15

TC = therapeutic climbing.

## Data Availability

A limited dataset is available from the corresponding author upon reasonable request.

## Supplementary Materials

The following supporting information can be downloaded at: https://www.mdpi.com/article/10.3390/healthcare12060674/s1.

## References

[1] WHO (2019). ICD-10: International Statistical Classification of Diseases and Related Health Problems 10th Revision.

[2] Tafti D., Ehsan M., Xixis K.L. (2022). Multiple Sclerosis.

[3] Kesselring J., Beer S. (2005). Symptomatic therapy and neurorehabilitation in multiple sclerosis. Lancet Neurol..

[4] Kwiatkowski A., Marissal J.P., Pouyfaucon M., Vermersch P., Hautecoeur P., Dervaux B. (2014). Social participation in patients with multiple sclerosis: Correlations between disability and economic burden. BMC Neurol..

[5] Salter A., Fox R.J., Tyry T., Cutter G., Marrie R.A. (2019). The association of fatigue and social participation in multiple sclerosis as assessed using two different instruments. Mult. Scler. Relat. Disord..

[6] DeSousa E.A., Albert R.H., Kalman B. (2002). Cognitive impairments in multiple sclerosis: A review. Am. J. Alzheimer’s Dis. Other Dement..

[7] Petracca M., Pontillo G., Moccia M., Carotenuto A., Cocozza S., Lanzillo R., Brunetti A., Brescia Morra V. (2021). Neuroimaging Correlates of Cognitive Dysfunction in Adults with Multiple Sclerosis. Brain Sci..

[8] Iodice R., Aceto G., Ruggiero L., Cassano E., Manganelli F., Dubbioso R. (2023). A review of current rehabilitation practices and their benefits in patients with multiple sclerosis. Mult. Scler. Relat. Disord..

[9] Leichtfried V., Franz Berghold H.B., Burtscher M., Domej W., Durrer B., Fischer R., Paal P., Schaffert W., Schobersberger W., Sumann G. (2015). Therapeutisches Klettern—Eine Extremsportart geht neue Wege. Alpin und Höhenmedizin.

[10] Frühauf A., Heußner J., Niedermeier M., Kopp M. (2021). Expert Views on Therapeutic Climbing—A Multi-Perspective, Qualitative Study. Int. J. Environ. Res. Public Health.

[11] Buechter R.B., Fechtelpeter D. (2011). Climbing for preventing and treating health problems: A systematic review of randomized controlled trials. Ger. Med. Sci..

[12] Liu S., Gong X., Li H., Li Y. (2022). The Origin, Application and Mechanism of Therapeutic Climbing: A Narrative Review. Int. J. Environ. Res. Public Health.

[13] Kern C., Elmenhorst J., Oberhoffer R. (2013). Effect of sport climbing on patients with multiple sclerosis—Hints or evidence?. Neurol. Rehabil..

[14] Gallotta M.C., Emerenziani G.P., Monteiro M.D., Iasevoli L., Iazzoni S., Baldari C., Guidetti L. (2015). Psychophysical benefits of rock-climbing activity. Percept. Mot. Ski..

[15] Mermier C.M., Robergs R.A., McMinn S.M., Heyward V.H. (1997). Energy expenditure and physiological responses during indoor rock climbing. Br. J. Sports Med..

[16] Rodio A., Fattorini L., Rosponi A., Quattrini F.M., Marchetti M. (2008). Physiological adaptation in noncompetitive rock climbers: Good for aerobic fitness?. J. Strength Cond. Res..

[17] Fruhauf A., Sevecke K., Kopp M. (2019). Current state of the scientific literature on effects of therapeutic climbing on mental health—Conclusion: A lot to do. Neuropsychiatr.

[18] Steimer J., Weissert R. (2017). Effects of Sport Climbing on Multiple Sclerosis. Front. Physiol..

[19] Velikonja O., Curic K., Ozura A., Jazbec S.S. (2010). Influence of sports climbing and yoga on spasticity, cognitive function, mood and fatigue in patients with multiple sclerosis. Clin. Neurol. Neurosurg..

[20] Tong A., Sainsbury P., Craig J. (2007). Consolidated criteria for reporting qualitative research (COREQ): A 32-item checklist for interviews and focus groups. Int. J. Qual. Health Care.

[21] NRZ Rosenhügel. https://www.nrz.at/.

[22] Nowell L.S., Norris J.M., White D.E., Moules N.J. (2017). Thematic analysis: Striving to meet the trustworthiness criteria. Int. J. Qual. Methods.

[23] Eppich W.J., Gormley G.J., Teunissen P.W., Nestel D., Hui J., Kunkler K., Scerbo M.W., Calhoun A.W. (2019). In-Depth Interviews. Healthcare Simulation Research: A Practical Guide.

[24] Kurtzke J.F. (1983). Rating neurologic impairment in multiple sclerosis: An expanded disability status scale (EDSS). Neurology.

[25] Kuckartz U. (2014). Qualitative Text Analysis: A Guide to Methods, Practice & Using Software.

[26] Gabler H. (2002). Motive im Sport: Motivationspsychologische Analysen und empirische Studien.

[27] Jeng B., DuBose N.G., Martin T.B., Silic P., Flores V.A., Zheng P., Motl R.W. (2023). An updated systematic review and quantitative synthesis of physical activity levels in multiple sclerosis. Am. J. Phys. Med. Rehabil..

[28] Alpenverein D., Alpenverein Ö., Südtirol A. (2016). Hoch Hinaus!.

[29] Gopal A., Bonanno V., Block V.J., Bove R.M. (2022). Accessibility to Telerehabilitation Services for People with Multiple Sclerosis: Analysis of Barriers and Limitations. Int. J. MS Care.

[30] Morimoto Y., Takahashi T., Sawa R., Saitoh M., Morisawa T., Kagiyama N., Kasai T., Dinesen B., Hollingdal M., Refsgaard J. (2022). Web Portals for Patients with Chronic Diseases: Scoping Review of the Functional Features and Theoretical Frameworks of Telerehabilitation Platforms. J. Med. Internet Res..

[31] Garber C.E., Blissmer B., Deschenes M.R., Franklin B.A., Lamonte M.J., Lee I.M., Nieman D.C., Swain D.P. (2011). American College of Sports Medicine position stand. Quantity and quality of exercise for developing and maintaining cardiorespiratory, musculoskeletal, and neuromotor fitness in apparently healthy adults: Guidance for prescribing exercise. Med. Sci. Sports Exerc..

[32] Watts P.B. (2004). Physiology of difficult rock climbing. Eur. J. Appl. Physiol..

[33] Gassner L., Dabnichki P., Langer A., Pokan R., Zach H., Ludwig M., Santer A. (2022). The therapeutic effects of climbing: A systematic review and meta-analysis. PMR.

[34] Rzepka M., Tos M., Boron M., Gibas K., Krzystanek E. (2020). Relationship between Fatigue and Physical Activity in a Polish Cohort of Multiple Sclerosis Patients. Medicina.

[35] Kalb R., Brown T.R., Coote S., Costello K., Dalgas U., Garmon E., Giesser B., Halper J., Karpatkin H., Keller J. (2020). Exercise and lifestyle physical activity recommendations for people with multiple sclerosis throughout the disease course. Mult. Scler..

[36] Dalgas U., Langeskov-Christensen M., Stenager E., Riemenschneider M., Hvid L.G. (2019). Exercise as Medicine in Multiple Sclerosis-Time for a Paradigm Shift: Preventive, Symptomatic, and Disease-Modifying Aspects and Perspectives. Curr. Neurol. Neurosci. Rep..

[37] Learmonth Y.C., Motl R.W. (2021). Exercise Training for Multiple Sclerosis: A Narrative Review of History, Benefits, Safety, Guidelines, and Promotion. Int. J. Environ. Res. Public Health.

[38] Halabchi F., Alizadeh Z., Sahraian M.A., Abolhasani M. (2017). Exercise prescription for patients with multiple sclerosis; potential benefits and practical recommendations. BMC Neurol..

[39] Reina-Gutiérrez S., Cavero-Redondo I., Martínez-Vizcaíno V., de Arenas-Arroyo S.N., López-Muñoz P., Álvarez-Bueno C., Guzmán-Pavón M.J., Torres-Costoso A. (2022). The type of exercise most beneficial for quality of life in people with multiple sclerosis: A network meta-analysis. Ann. Phys. Rehabil. Med..

[40] Sandroff B.M., Motl R.W., Scudder M.R., DeLuca J. (2016). Systematic, Evidence-Based Review of Exercise, Physical Activity, and Physical Fitness Effects on Cognition in Persons with Multiple Sclerosis. Neuropsychol. Rev..

