# Supplementary material for: Perceptions of Therapeutic Climbing for Patients with Multiple Sclerosis in Neurorehabilitation: A Qualitative Study
Source: Healthcare (Basel). 2024 Mar 17;12(6):674. doi: 10.3390/healthcare12060674 (PMC10970378; doi:10.3390/healthcare12060674)
Supplement: Supplementary file 1 [file healthcare-12-00674-s001.zip › File S1.pdf]

File S1. Question Schedules for Interviews with Specific Study Groups.

Question schedule – Patients.

1. **Training modalities:** You participated in therapeutic climbing. How was this therapy for you?
    a. **Exercise selection/content:** Can you remember an exercise that you particularly liked? Could you please describe it?
    b. **Dosage:** How often and how long did you train on the climbing wall? Did you find that appropriate, or would you have liked more or less training time and units?
2. **Motivation:** When you think of therapeutic climbing, what do you think of?
    a. Do you have any feelings about therapeutic climbing?
    b. What did you like about therapeutic climbing? Why?
    c. **Volition:** When things got tiring, what kept you going?
    d. **Special features:** In your opinion, what distinguishes therapeutic climbing from other forms of therapy?
3. **Effects:** What physical/mental changes did you notice that you can attribute to therapeutic climbing?
    a. **Physical effects:** Various effects on the body are attributable to climbing. I will give you some examples and ask you to assess whether you have noticed any changes in this regard.
        i. Did climbing affect your **endurance**? If so, to what extent?
        ii. Did climbing affect your **mobility**? If so, to what extent?
        iii. Did climbing affect your **strength**? If so, to what extent?
        iv. Did climbing affect your **coordination**? If so, to what extent?
        v. Did climbing affect your **balance**? If so, to what extent?
    b. **Psychological effects:** Climbing is said to have various effects on the mind. I will give you some examples and ask you to assess whether you have noticed any changes in this regard.
        i. Did climbing affect your **body awareness**? If so, to what extent?
        ii. Did climbing affect your **existing fears**? If so, to what extent?
        iii. Did climbing affect your **self-confidence**? If so, to what extent?
    c. **Fatigue:** Fatigue is a common symptom in patients with multiple sclerosis. If you are also affected, could you please tell me if you notice any changes in this regard? If so, what are these changes? Now, could you please distinguish between the effects on fatigue during therapeutic climbing, immediately after, and considered throughout the rehabilitation stay?
    d. **Complications:** Did you notice any adverse effects? Did you have any pain during or after therapeutic climbing?
4. **Safety:** Were there situations in which you were afraid?
    a. How safe did you feel?
    b. Why were you afraid?
    c. How did you deal with the fear?
5. **Applicability:** How suitable was therapeutic climbing for you?
    a. **Subjective perception:** Did you feel that therapeutic climbing was helping you? How did you experience it?
    b. **Physical requirements:** What physical requirements did you need to participate in therapeutic climbing?
    c. **Restrictions:** Did you notice any limitations in performing the exercises? To what extent did the restrictions affect how you felt? Why did this change in condition occur?

6. **Other:** Is there anything else you would like to add to this discussion, or do you have any questions for me?

Question schedule – Therapists.

1. **Training modalities:** You use therapeutic climbing for patients with multiple sclerosis. What does the therapy look like in concrete terms?
    a. **Exercise selection/training structure:** Could you please describe the training structure? Is there an exercise you find particularly useful that you can describe to me?
    b. **Dosage:** How long and how often do you think patients with multiple sclerosis should train on the climbing wall?
2. **Motivation:** Why do you train with patients with multiple sclerosis on the climbing wall?
    a. What do you think distinguishes therapeutic climbing, or how is it different from other forms of therapy?
3. **Effects:** What physical/psychological effects do you observe in patients with multiple sclerosis that you can attribute to therapeutic climbing?
    a. **Fatigue:** Fatigue is a common symptom in patients with multiple sclerosis. What changes in this regard do you notice in patients during therapeutic climbing? Can you please distinguish between effects during the climbing sessions, immediately after, and throughout the rehabilitation stay?
    b. **Complications:** Do complications occur when using therapeutic climbing in patients with multiple sclerosis? Do patients report shoulder or neck pain?
4. **Safety:** How safe is therapeutic climbing?
    a. Have there been dangerous situations or even injuries to patients with multiple sclerosis in your presence during therapeutic climbing?
5. **Applicability:** Which patients with multiple sclerosis can participate in the therapeutic climbing intervention?
    a. **Physical requirements:** What physical requirements do patients with multiple sclerosis need to participate in therapeutic climbing?
6. **Exclusion and termination criteria:** Which patients with multiple sclerosis cannot participate in the therapeutic climbing intervention?
    a. **Contraindications:** Are there any contraindications for therapeutic climbing? What were the reasons for **discontinuing or stopping** therapeutic climbing?
    b. **Restrictions:** Are there any afflictions that prevent patients with multiple sclerosis from performing individual therapeutic climbing exercises?
7. **Other:** Is there anything else you would like to add to this discussion, or do you have any questions for me?
